# In silico exploration of the genomic repertoire of Iranian aquatic bacteria: Prophage carriage, bioactive compound potential, CRISPR-Cas immunity, and integrated defensive-metabolic islands

**DOI:** 10.1016/j.bbrep.2026.102452

**Published:** 2026-01-28

**Authors:** Mohammadreza Rahimian, Mohammad Aghazadeh-Soltan-Ahmadi, Bahman Panahi

**Affiliations:** a Department of Biology, Faculty of Basic Sciences, University of Maragheh, Maragheh, Iran; b Department of Animal Biology, Faculty of Natural Science, University of Tabriz, Tabriz, Iran; c Department of Genomics, Branch for Northwest & West Region, Agricultural Biotechnology Research Institute of Iran (ABRII), Agricultural Research, Education and Extension Organization (AREEO), Tabriz, Iran

**Keywords:** Genome mining, Prophage, Secondary metabolites, Bacteriocins, Metabolic-defense islands, Biosynthetic gene clusters (BGCs), Extreme environments

## Abstract

The unique and underexplored aquatic ecosystems of Iran represent a significant reservoir of microbial diversity. This study presents the first comprehensive genomic survey of 38 native Iranian bacterial strains from hypersaline lakes and wetlands, integrating in silico analyses of their secondary metabolome, bacteriocin potential, resident prophages, and genomic architecture. Our genome mining revealed a prolific capacity for secondary metabolite production, identifying dozens of biosynthetic gene clusters (BGCs). Ectoine biosynthesis was ubiquitous, underscoring its role as a key osmoprotectant, while diverse BGCs for terpenes, polyketides, and hybrid metabolites were also prevalent. Concurrently, we identified a wide array of ribosomally synthesized and post-translationally modified peptides (RiPPs), including known bacteriocins. Furthermore, we characterized eight high-quality prophages integrated within these genomes, encoding auxiliary genes such as carbohydrate-active enzymes (CAZymes) and putative *anti*-CRISPR (ACR) proteins. The bacterial hosts themselves were equipped with robust defense systems, with CRISPR-Cas loci, predominantly Type I, detected in most strains. Crucially, we identified multi-functional genomic islands that physically link BGCs with defense systems (e.g., CRISPR-Cas, restriction-modification) and prophage regions. We propose the “Fortress Hypothesis” to explain this architecture, wherein the co-localization of metabolite production and defense machinery protects metabolic investment against phage predation and genetic loss. This integrative genomic arrangement highlights a sophisticated co-evolutionary strategy for survival in extreme environments. Our findings position these indigenous bacteria as a promising genetic repository for discovering novel bioactive compounds, enzymes, and biotechnological tools, with implications for antibiotic discovery, CRISPR modulation, and understanding adaptive microbial genomics in extreme niches.

## Highlights

• Discovery of 23 multi-functional genomic islands in bacterial genomes.
• Proposed ‘Fortress Hypothesis' explains co-localized defense & biosynthesis.
• Eight prophages encode auxiliary CAZymes and *anti*-CRISPR proteins.
• CRISPR-Cas systems detected in 34 of 38 strains, predominantly Type I.
• Diverse BGCs identified, including ubiquitous ectoine biosynthesis.

## 1 Introduction

Having originated over 3.5 billion years ago, marine life has evolved sophisticated survival mechanisms for a hostile environment. While bioactive compounds from terrestrial organisms are well-known, the unique adaptations of diverse marine life are expected to yield a wide array of unique bioactive molecules [1]. Among these, microbial secondary metabolites are non-essential small compounds that play vital ecological roles [2]. They mediate interactions by providing selective advantages like antibiotic action, which enhances a microbe's ability to compete and survive in its environment [3].

The World Health Organization (WHO) considers antibiotic resistance a critical global health threat because it undermines the treatment of common infectious diseases [4,5]. Bacteriocins, which are antimicrobial peptides produced by bacteria, have generated significant interest as potential alternatives to conventional antibiotics. These compounds are synthesized by ribosomes and can target a narrow or broad range of microbes [6]. Therefore, as a class of secondary metabolites, bacteriocins not only serve the previously described ecological roles but also hold great promise as a source for new antibiotics.

Bacteriophages (phages), the viruses that infect bacteria, play pivotal roles in shaping microbial evolution, ecology, and dynamics. Phages exist in diverse environmental niches, including water, extreme-conditioned ecosystems, and soil [7]. The high prevalence and infectivity of phages against bacteria underscores the phages' undeniable effect on their host and ecosystem [8]. Their integration into bacterial genomes as prophages contributes not only to horizontal gene transfer but also to phenotypic diversification, including virulence, antibiotic resistance, and environmental adaptability [9]. With the growing availability of whole-genome sequencing data, in silico prophage mining has become a powerful strategy to uncover hidden viral elements in bacterial genomes [10,11].

Due to high accessibility to high-throughput sequence data on the internet and the emergence of various types of bioinformatic tools, data mining approaches have become a very reliable approach to investigate bacterial genomes and unveil the hidden world of phages in them [12]. The study of phage-host relationships holds profound importance for microbiology, ecology, medicine, and biotechnology [13]. Understanding phage-host dynamics is crucial for revealing fundamental principles of microbial ecology [14]. Furthermore, the study of phage-host interplay uncovers mechanisms of molecular evolution and coevolution, which are essential for understanding the emergence of new bacterial strains and the evolution of phage infectivity [15]. In this case, receptor-binding proteins (RBPs), auxiliary metabolic genes (AMGs), and carbohydrate-active enzymes (CAZymes) are considered essential factors to study phage-bacteria interactions.

The initial step in phage infection is the attachment to the host bacterium's surface. This binding event is facilitated by RBPs on the phage that specifically recognize and adhere to complementary receptors on the bacterium. This highly specific RBP-receptor interaction is essential for successful infection and is the primary determinant of the phage's host specificity [16]. Furthermore, the relationship between prophages and their bacterial hosts can influence ecological dynamics under certain conditions. This effect is associated with prophages that harbor AMGs. Through these AMGs, prophages have the ability to alter the host's metabolic pathways, thereby improving both phage replication efficiency and the overall fitness of the infected bacterial cells [17]. Another aspect of phage-host dynamics is the role of phage-derived CAZymes. These enzymes help the host bacteria to digest diverse carbon sources and persist in challenging conditions [18].

One of the important aspects of phage-bacteria interaction is the emerging phage defense systems in bacteria. The study of phage defense systems in bacteria is critically important because bacteria are under constant threat from phages [19]. These defense systems represent a broad and diverse arsenal that bacteria have evolved to resist phage infection, involving various mechanisms to prevent phage attachment, replication, or to induce cell death upon infection [20,21]. Understanding these mechanisms is essential for fundamental insights into microbial ecology and evolution [22]. The CRISPR (clustered regularly interspaced short palindromic repeats)—Cas system plays a pivotal role in phage-bacteria interaction as a part of the bacterial defense system against phages [23,24].

The CRISPR-Cas system stands at the forefront of evolutionary studies on phage-host interactions, offering deep insights into the ongoing arms race between bacteria and their viral predators [23,24]. As an adaptive immune mechanism, CRISPR-Cas enables bacteria to acquire memory of past viral infections by integrating fragments of phage DNA (spacers) into their own genome, thus granting targeted resistance against recurrent attacks. This dynamic not only protects bacterial populations but also exerts strong evolutionary pressure on phages, compelling them to evolve strategies such as spacer escape mutations and the deployment of *anti*-CRISPR proteins [25,26]. Studying CRISPR-Cas systems in this evolutionary context illuminates the co-evolutionary processes shaping both microbial ecology and genetic diversity. Long-term laboratory and environmental studies reveal how the back-and-forth of bacterial defense and phage counter-defense leads to rapid genome evolution in both parties, providing a powerful model for understanding molecular adaptation, genetic innovation, and population structure in natural microbial communities [27,28].

Iran, with its diverse aquatic and water-related ecosystems, offers a largely untapped reservoir of bacterial diversity [29]. However, limited studies have explored the phage content of native Iranian bacterial isolates from these habitats. Understanding the prophage landscape of these bacteria is critical for phage biology and co-evolutionary dynamics. In this study, we analyzed the genomes of a collection of aquatic bacterial strains to identify integrated prophages and prophage-like elements. By focusing on these unique, regionally isolated genomes from water-based niches, we aim to expand our knowledge of the phage biodiversity associated with underexplored bacterial populations and to contribute new insights into the viral elements that reside within them.

Based on the observed genomic architecture, we hypothesize that these bacteria have evolved multi-functional genomic islands as an adaptive strategy to protect metabolically costly secondary metabolite production in extreme and competitive environments. We further propose that this co-localization may facilitate co-regulation, ensuring that defense mechanisms are activated concurrently with metabolite synthesis, thereby optimizing survival and resource allocation under stress. To test this hypothesis and expand our knowledge of phage biodiversity, we analyzed the genomes of a collection of aquatic bacterial strains to identify not only integrated prophages and prophage-like elements but also to map the genomic architecture linking these elements with biosynthetic and defense systems.

## 2 Methods

### 2.1 Genomic data set

The complete list of native bacterial strains was retrieved from the official website of the Iranian Biological Resource Center (IBRC). A total of 38 genomes belong to 23 strains were downloaded from the National Center for Biotechnology Information (NCBI) (https://www.ncbi.nlm.nih.gov/) gene bank on July 5, 2025. The characteristics of the investigated bacteria are summarized in Table 1.

**Table 1:** Summary information of investigated bacteria.

Bacteria name	Gram	Number of strains	Habitat
*Aliicoccus persicus*	+	4	A brine sample from a Hypersaline lake brine
*Aliidiomarina iranensis*	–	1	Coastal-marine wetland
*Aliidiomarina sedimenti*	–	1	A sediment sample from the coastal-marine wetland
*Alteribacillus bidgolensis*	+	2	Hypersaline lake water
*Alteribacillus iranensis*	+	1	Hypersaline lake mud
*Alteribacillus persepolensis*	+	1	Water of the hypersaline lake
*Aquibacillus halophilus*	+	1	Hypersaline lake water
*Halomonas lysinitropha*	–	1	Hypersaline wetland
*Lentibacillus persicus*	+	1	Hypersaline lake water
*Limimonas halophila*	–	1	The saline mud of the hypersaline lake
*Marinobacter azerbaijanicus*	–	1	Hypersaline lake water
*Marinobacter iranensis*	–	1	Hypersaline lake water
*Marinobacter persicus*	–	6	A brine sample of the hypersaline lake
*Oceanimonas* sp. GK1	–	1	Marine wetland
*Oceanobacillus limi*	+	1	A mud sample of the hypersaline lake
*Oceanobacillus longus*	+	1	Hypersaline lake brine
*Ornithinibacillus halophilus*	+	1	Hypersaline lake water
*Piscibacillus halophilus*	+	3	Water of the hypersaline lake
*Salininema proteolyticum*	+	2	Wetland soil
*Salinispirillum marinum*	–	2	Coastal-marine wetland water
*Salinithrix halophila*	+	1	Hypersaline wetland soil
*Salinivibrio proteolyticus*	–	3	Hypersaline lake water
*Thalassobacillus cyri*	+	1	Water of the hypersaline lake

### 2.2 Identification of putative secondary metabolite and bacteriocin gene clusters

To conduct a comprehensive analysis of secondary metabolites, bacterial genomes were screened for biosynthetic gene clusters (BGCs) using two specialized tools.

First, the antiSMASH v8.0.4 tool in strict mode was employed to systematically identify a broad range of secondary metabolite gene clusters [30]. Subsequently, a more targeted search for a specific class of these metabolites (bacteriocins) was performed using the BAGEL4 v1.2 web server with its default parameters [31].

### 2.3 Prophage identification

Prophage detection in the bacterial genome was performed using PHASTEST (PHAge Search Tool with Enhanced Sequence Translation) v1.0.1 [32] and a machine learning-based tool, VirSorter2 v2.2.4, with choosing minimum sequence length of 500 and a minimum score of 0.4 [33]. The PHASTEST tool categorizes predicted prophages into three distinct completeness classes (incomplete (score <70), questionable (70 ≤ score ≤90), and intact (score >90)) by evaluating the presence and organization of essential prophage structural and functional modules. This includes head, capsid, tail, integrase, and lysis-related genes.

Intact prophage sequences from the PHASTEST tool and all prophages identified by VirSorter2 were then thoroughly evaluated using CheckV v1.5 with default parameters to identify any host contamination within the prophage sequences and to assess their completeness and quality [34]. Only prophages with high quality, defined as having more than 90 % completeness and less than 5 % of contamination, were selected for further analyses.

### 2.4 Protein annotation, lifestyle prediction, and taxonomic classification

The PhaBOX v2.0 web service (https://phage.ee.cityu.edu.hk/phabox) was used to assess lifestyle prediction and taxonomic classification of identified prophages [35]. This web-based platform uses PhaTYP [36] and PhaGCN [37], respectively, for this purpose. Also, the PhageScope web tool (https://phagescope.deepomics.org/) v1.3 was used to annotate the predicted prophages [38]. All these tools were used based on their default parameters.

### 2.5 Average Nucleotide Identity (ANI), phylogenetic, and comparative genomic analysis

The genomic similarity of high-quality prophages and bacterial genomes was evaluated using Average Nucleotide Identity (ANI) analysis through the FastANI tool v1.3 [39] provided by the Galaxy Europe web platform (https://usegalaxy.eu/) v25.0.4.dev0 [40]. ANI results visualized by Morpheus (https://software.broadinstitute.org/morpheus). Phylogenetic analysis was performed on selected prophages, producing a proteomic tree and determining their placement within the viral tree of life using the ViPTree web server v4.0 [41]. This tool uses an alignment-free, whole-proteome phylogeny method based on genome-wide similarities to construct phylogenetic trees for viruses.

Furthermore, based on BLASTn alignment results, complete prophages showing the highest sequence similarity to publicly available phage sequences in the NCBI database were selected for comparative genomics analysis with ViPTree. Any phage sequences from BLASTn analyses with unknown host, lifestyle, and taxonomy were subjected to host, lifestyle, and taxonomy assignment via CHERRY [42] and previously mentioned tools. In addition, prophages with very high ANI scores underwent further comparative genome-level analysis to examine their close genetic relationships using the same tool.

### 2.6 AMG, CAZyme, and RBP identification

Putative AMGs within prophages were identified following the approach described by Luo et al., with minor modifications [43]. In summary, all phage proteins initially labeled as unsorted or hypothetical were collected and subsequently re-annotated using eggNOG-mapper version 2 [44] and InterProScan v107.0 [45]. Proteins that were annotated and predicted to be involved in metabolic pathways were then subjected to KEGG annotation using BlastKOALA v3.1 [46]. The prediction of CAZymes was performed with the dbCAN3 tool v14, accessible at https://bcb.unl.edu/dbCAN2/.

The identification of RBPs in predicted prophages was conducted using the PhageRBPdetection tool v4 [47]. All unclassified proteins (such as hypothetical or unknown proteins) which is identified as RBP were subjected to InterProScan and BLASTp analysis against RefSeq, UniProtKB reference proteomes, and the Swiss-Prot database under stringent parameters (e-value ≤0.0001, identity ≥100 %, coverage ≥100 %).

### 2.7 CRISPR, defense, and anti-defense system analysis

All chosen prophages, along with 38 bacterial strains, were screened for the presence of CRISPR-Cas systems using the CRISPRCasFinder [48] and CRISPRCasMeta tools within the CRISPR-Cas++ web service (https://crisprcas.i2bc.paris-saclay.fr/) v1.1.2. All these tools were run with default parameters. The identified spacers were then aligned against the predicted prophages using BLAST, requiring perfect matches across the entire spacer length with zero mismatches and an e-value threshold of 0.0001 or lower [18]. Frequency of CRISPR, spacers, and Cas protein types among bacterial genomes visualized using the Circos data visualization tool [49].

Additionally, proteins classified as unsorted or hypothetical from the selected prophages were examined for potential *anti*-CRISPR (ACR) proteins utilizing the AcrHub prediction platform (as available at https://pacrispr.erc.monash.edu/AcrHub/) by utilizing all three available tools, including PaCRISPR, AcRanker, and HMM-based predictor [50]. Furthermore, both the selected prophages and all bacterial strains were evaluated for other defense and anti-defense mechanisms beyond CRISPR and ACR proteins, employing the DefenseFinder tool v2.0.0 [51].

### 2.8 Multi-functional genomic island analysis

A spatial proximity-based method was implemented to detect and characterize functional genomic islands that combine metabolic, defensive, and prophage components. This approach involved mapping the physical clustering of key genomic features, such as BGCs, defense systems (including CRISPR-Cas arrays, defense, and anti-defense systems), and integrated prophage regions within bacterial genomes. Genomic islands were defined according to two criteria: first, all relevant elements had to be located on the same contig; and second, the maximum intervening distance between any two adjacent elements could not exceed 50 kilobase pairs. This distance threshold was adopted based on earlier research indicating that functional interactions between defense-related genetic elements commonly occur within this genomic range [52].

Three distinct genomic island models, including “defensive-metabolic islands,” comprising both defense systems and BGCs; “prophage-defensive-metabolic islands,” containing prophages, defense systems, and BGCs; and “prophage-metabolic islands,” which included prophages and BGCs but lacked dedicated defense systems, were used for categorization. Instances where the genomic coordinates of different elements overlapped were carefully recorded.

## 3 Results

### 3.1 Identified secondary metabolite gene cassettes

#### 3.1.1 Identification of single-candidate BGCs

**Fig. 1:**
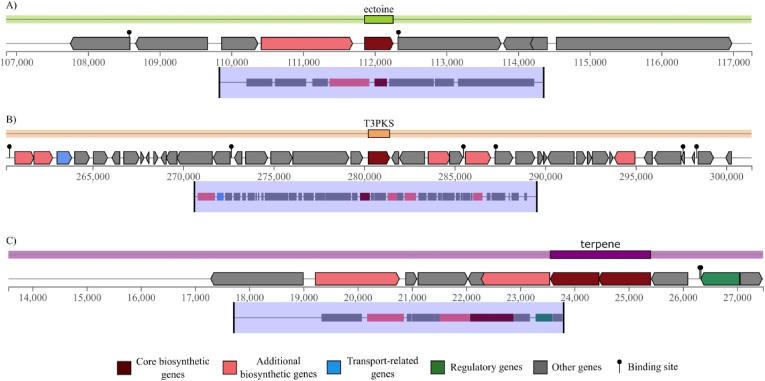
Examples of identified secondary metabolites in bacterial genomes. (A) Ectoine gene cluster from *Aliidiomarina iranensis*. (B) T3PKS gene cluster from *Oceanobacillus longus*. (C) Terpene from *L. halophila*.

The genome mining effort identified a diverse array of single biosynthetic gene cassettes each dedicated to the production of a single class of secondary metabolite. The most prevalent type was the ectoine biosynthetic gene cluster, identified 43 times, underscoring its fundamental role as a widespread compatible solute (Fig. 1). Betalactone clusters were identified on 18 occasions. Terpene and Type III Polyketide Synthase (T3PKS) clusters were also common, each being detected 17 times, while NI-siderophore clusters were found 16 times. Less abundant but notable clusters included those for hydrogen-cyanide (4), hserlactone (3), redox-cofactor (2), melanin (2), and resorcinol (2). Several unique clusters were observed singularly, including those for arylpolyene, Type I Polyketide Synthase (T1PKS), and phosphonate. A comparative analysis across specific isolates revealed that no secondary metabolites were identified in the genomes of *A. persicus* isolate 6019 and isolate Pva5IMA98y. In contrast, the genome of *L. halophila* contained the highest number of metabolite gene clusters, with a total of nine distinct cassettes identified.

#### 3.1.2 Hybrid candidate clusters

**Fig. 2:**
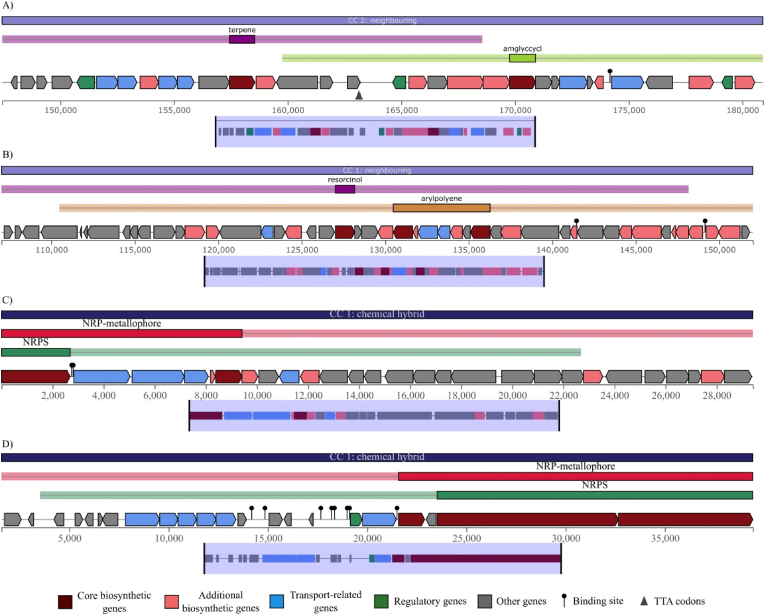
Examples of identified hybrid secondary metabolites in bacterial genomes. (A) Hybrid amglyccycl and terpene cassette from *Salininema proteolyticum* strain IBRC-M 10908. (B) Hybrid arylpolyene and resorcinol cassette from *M. persicus* strain UTICA-S1B9. (C and D) Two hybrid NRP-metallophore and NRPS cassettes from *M*. *iranensis*.

The investigation also uncovered several hybrid BGCs, which are defined as genomic loci encoding for the biosynthesis of multiple, distinct classes of secondary metabolites. A notable finding was the identification of six hybrid clusters combining arylpolyene and resorcinol pathways, all of which were located within genomes of *M. persicus* and were physically neighbouring. Furthermore, two separate hybrid clusters were identified, each pairing a nonribosomal peptide synthetase (NRPS) cluster with a nonribosomal peptide-derived metallophore (NRP-metallophore) cluster; both of these were found exclusively in *M. iranensis* and were classified as chemical hybrids. Additional neighbouring hybrid clusters included one instance each of azole-containing-post-translational modified peptides (RiPP) with betalactone, T3PKS with ectoine, and T3PKS with terpene, as well as two instances of amglyccycl paired with terpene. Representative examples of these hybrid cluster architectures are illustrated in Fig. 2.

### 3.2 Identified bacteriocins

**Fig. 3:**
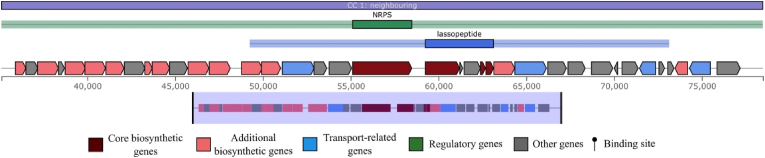
A hybrid gene cassette contains NRPS and lassopeptide identified from *A*. *halophilus*.

The BAGEL4 analysis identified two main categories of bacteriocins. The first category consisted of BGCs for which a specific class of bacteriocin was identified, but no core biosynthetic proteins were definitively called. These putative bacteriocins were dominated by Sactipeptides (14 BGCs), followed by LAPs (Lantibiotic-like peptides, 5 BGCs), and smaller numbers of Lasso_peptides, Bottromycin, Microcin, Lanthipeptide_class_IV, and an UviB. Moreover, a hybrid gene cassette including NRPS and lassopeptide identified in *A. halophilus* (Fig. 3).

**Fig. 4:**
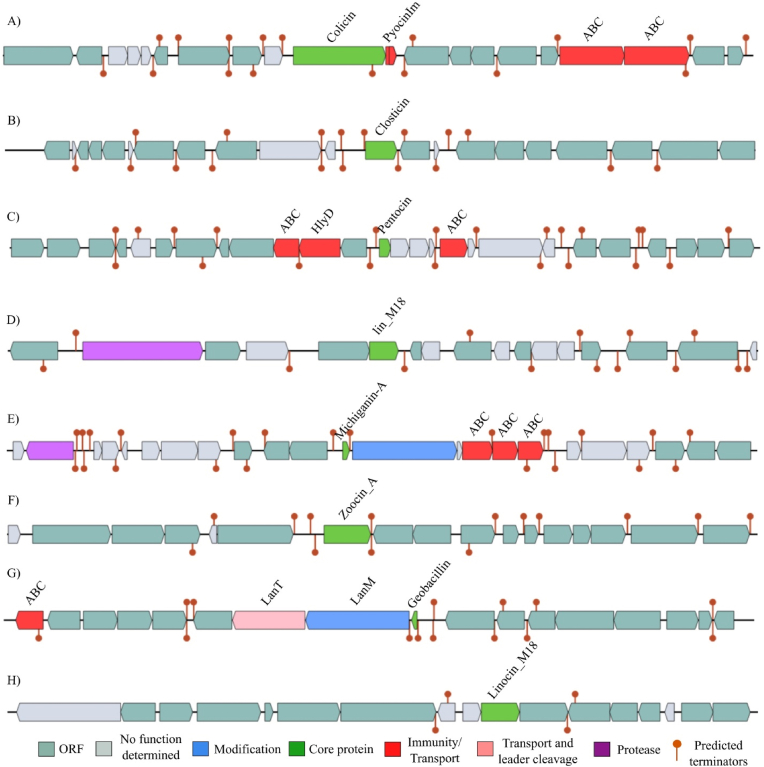
Examples of bacteriocin gene cassettes that contain core protein identified in bacterial genomes. (A) Colicin from *Salinivibrio proteolyticus* strain TGB10. (B) Closticin_574 from *S*. *halophila*. (C) Butyrivibriocin_AR10 from *S*. *halophila*. (D) A putative bacteriocin from *S*. *proteolyticum* strain IBRC-M 10908. (E) Michiganin-A from *S*. *proteolyticum* strain IBRC-M 10908 Contig1. (F) Zoocin_A from *Piscibacillus halophilus* isolate bin41. (G) Geobacillin_II from *Oceanobacillus limi*. (H) Linocin_M18 from *M*. *persicus* strain UTICA-S1B9.

The second, more definitive category included bacteriocins for which the core protein was successfully identified (Fig. 4). Among these, we detected BGCs for LINOCIN_M18 (3 BGCs), Zoocin_A (3 BGCs), Michiganin-A (2 BGCs), and single instances of Colicin, Closticin_574, Butyrivibriocin_AR10, geobacillin_II, and a putative_Bacteriocin_family_protein.

This genomic potential was corroborated by analysis using the antiSMASH tool, which identified additional BGCs encoding ribosomally synthesized and post-translationally modified peptides (RiPPs). The antiSMASH results notably included multiple BGCs for lanthipeptide-class-ii (2 BGCs), lanthipeptide-class-iii (4 BGCs), and lanthipeptide-class-iv (2 BGCs), which align with and expand upon the 'LAPs' and ‘Lanthipeptide_class_IV’ categories noted by BAGEL4. Furthermore, antiSMASH detected several clusters for azole-containing-RiPPs (6 BGCs, one hybrid with a betalactone). It also confirmed the presence of the NRPS, lassopeptide hybrid and an additional lassopeptide cluster, as well as a ranthipeptide BGC.

All analyzed genomes collectively encoded a total of 46 unique bacteriocin BGCs. Among these, *S. halophila* was a notable producer, containing 12 BGCs, half of which (6 BGCs) were dedicated to bacteriocin synthesis. After consolidating and deduplicating the results from both the BAGEL4 and antiSMASH analyses, each of the 46 identified bacteriocins represented a distinct, non-redundant genetic determinant.

### 3.3 Prophage identification

To identify the putative prophages, in total, 38 bacterial genomes were downloaded. Using the PHASTEST tool, a total of 37 prophage sequences were predicted from 16 strains. Among 37 prophage sequences, 15, 10, and 12 sequences were determined as intact, questionable, and incomplete sequences, respectively. The identified intact prophages have an average contig length of 34.85 kbp. The shortest contig length was 19.3 kbp, while the longest was 48.2 kbp. Moreover, VirSorter2 identified 85 prophages. The average size of the prophages identified by VirSorter2 was 31.75 kbp, with the longest contig 105.28 kbp, and the shortest contig was 1.40 kbp. Among the predicted prophages, eight unique sequences met quality standards, which were chosen for further analysis.

### 3.4 Bacterial genomes and prophages similarity

**Fig. 5:**
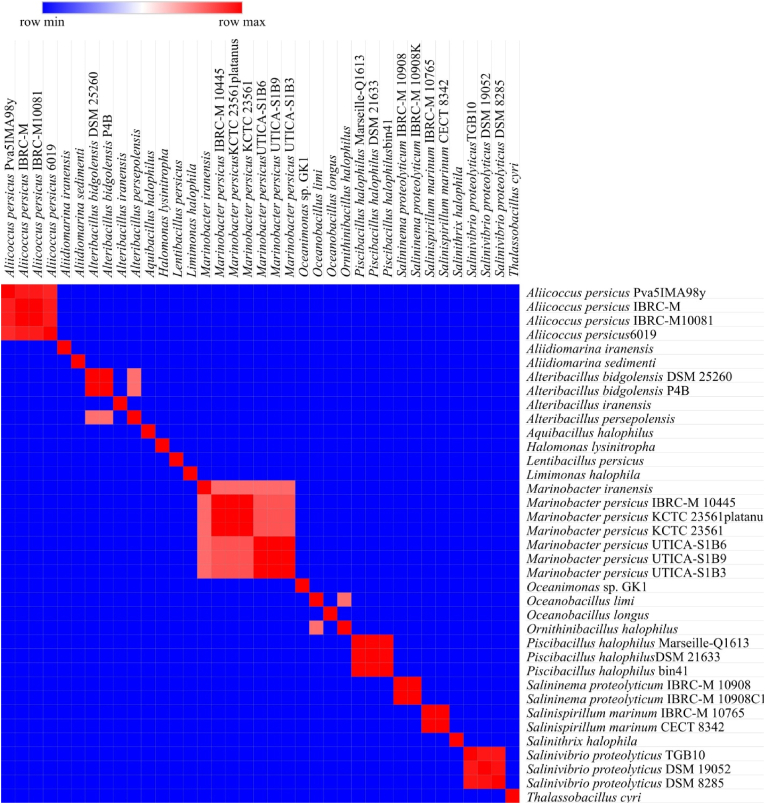
ANI heatmap for 37 bacterial strains.

The pairwise FastANI comparisons of 38 bacterial strains produced values between 75.89 % and 99.9984 % (Fig. 5). Within each species group ANI remained ≥78 %, reaching 99.98 % among the four *A*. *persicus* strains, 99.37 % among the three *P. halophilus* strains, 96.85 % among the three *S. proteolyticus* strains, 99.97 % between the two *S. proteolyticum* strains, 99.99 % between the two *S. marinum* strains, and 99.99 % between the six *M. persicus* strains. Between species ANI dropped below 78.3 %, with the lowest value of 78.1 % recorded between *O*. *limi* and *O. halophilus*.

Eight prophage sequences were compared similarly. Self-comparisons gave 100 % identity across all the prophages. The only exception was Ph_Salpro1 versus Ph_Salpro4 at 99.998 %. No prophage shared similarity with a prophage from a different host species.

### 3.5 Prophages characteristics

Based on the comprehensive annotation analyses, the eight selected phages represent four taxonomic lineages, including *Caudoviricetes*, *Arquatrovirinae*, Streptomyces phage Chymera, and Bielevirus phiO18P (Table 2). All of the prophages show a temperate lifestyle except for Ph_Sapr1, which was identified as virulent. Across the data set, the mean genome length is 50.4 kbp, ranging from the 36.9 kbp of Ph_Salpro3 to the 60.6 kbp of Ph_Salpro2, while the average GC content is 56.36 % and spans from 39.8 % in Ph_Alpe to 65.6 % in Ph_Salpro3. Ph_Salpro2 simultaneously possesses the largest genome and the highest number of genes (75 in total). Ph_Ocea carries the fewest replication genes, only one, and Ph_Salpro3 combines the shortest genome with the highest GC content observed. Ph_Sapr1 is unique among the set in harbouring five lysis genes. Ph_Alpe stands out for its investment in host-interaction functions, leading the integration, regulation, packaging, and replication categories with five genes except for replication (seven genes). Circular genome maps illustrating the overall architecture of each phage are presented in Supplementary Material 2.

**Table 2:** Summary of prophages characteristics.

Prophage name	Host	Length (kbp)	GC content (%)	Lifestyle	Taxonomic rank
Ph_Alpe	*A. persicus*	43.5	39.81	temperate	*Caudoviricetes*
Ph_Ocea	*Oceanimonas* sp. GK1	38.4	58.25	temperate	Bielevirus phiO18P
Ph_Salpro1	*S. proteolyticum* IBRC-M 10908	60.1	62.84	temperate	*Arquatrovirinae*
Ph_Salpro2	*S. proteolyticum* IBRC-M 10908	60.6	63.05	temperate	*Arquatrovirinae*
Ph_Salpro3	*S. proteolyticum* IBRC-M 10908	36.9	65.63	temperate	Streptomyces phage Chymera
Ph_Salpro4	*S. proteolyticum* IBRC-M 10908K	60.1	62.84	temperate	*Arquatrovirinae*
Ph_Sapr1	*S. proteolyticus* 19052	44.2	51.82	virulent	*Caudoviricetes*
Ph_Sapr2	*S. proteolyticus* TGB10	59.5	46.69	temperate	*Caudoviricetes*

### 3.6 Phylogenetic analysis

**Fig. 6:**
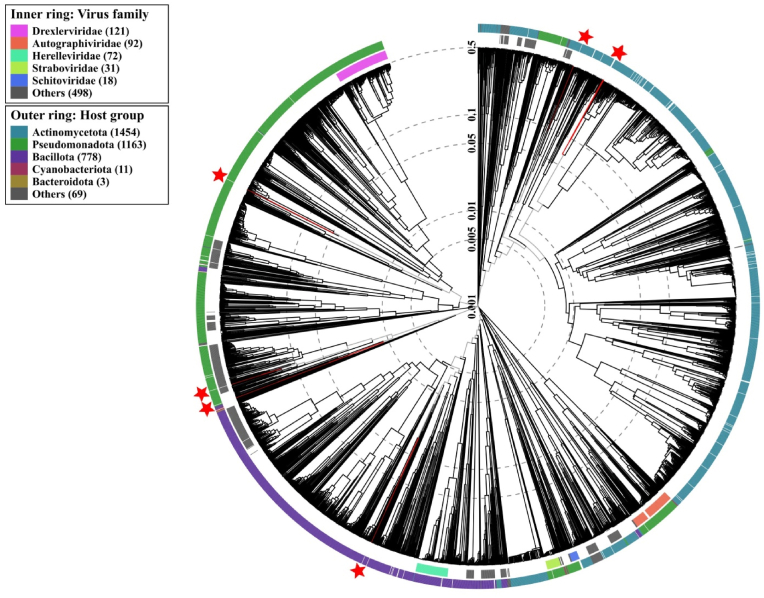
VipTree phylogenomic analysis of prophage sequences.

The phylogenetic analysis generated through the VipTree tool revealed that the group formed by Ph_Salpro1, Ph_Salpro2, and Ph_Salpro4 was positioned within a clade of diverse *Streptomyces* phages (Fig. 6). In another cluster, Ph_Sapr1 were positioned in close association with Vibrio phages that primarily infect *Vibrio* species. The phylogeny also showed that Ph_Ocea grouped with phages that can infect *Aeromonas* and *Pasteurella*. The analysis further revealed that Ph_Sapr2 shares a close phylogenetic relationship with *Shigella* phages. The group containing Ph_Alpe emerged in association with phages related to *Paenibacillus* infecting phages.

### 3.7 Comparative genomic analysis

Comparative genomic analysis using ViPTree provided further insights into the evolutionary relationships between the identified prophages and their closest reference genomes. Based on BLASTn alignments, prophages with the highest sequence similarity to publicly available phages in the NCBI database were selected, and additional classification was performed for those lacking host, taxonomy, or lifestyle information.

**Fig. 7:**
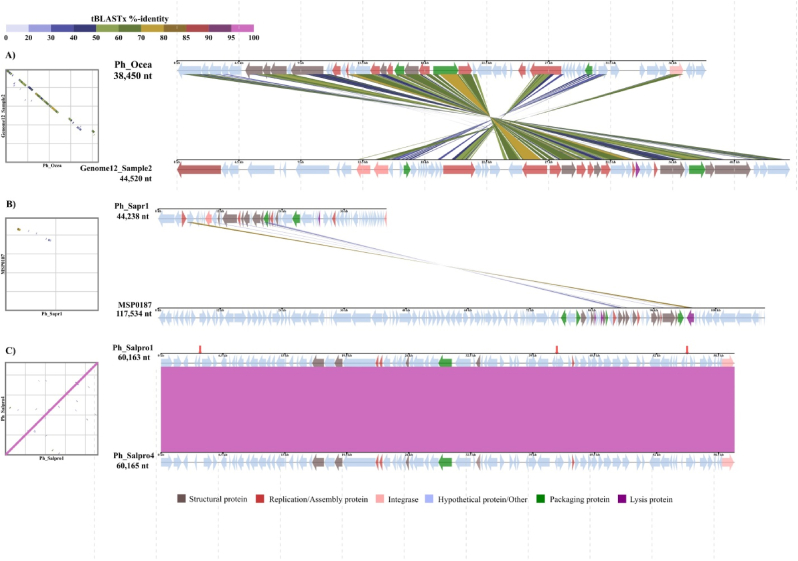
Comparative genomic analysis of prophage clusters. (A) Alignment of Ph_Agbu with its closest relative (MAG MSP0043), showing regions of synteny and rearrangement. (B) Near-identical genomes of Ph_Haka2 and Ph_Haka3 demonstrate high collinearity. (C) Genomic alignment of Ph_Salpro1 and Ph_Salpro4 reveals extensive collinearity within the cluster.

In the Ph_Salpro cluster, Ph_Salpro1, Ph_Salpro2, and Ph_Salpro4 showed limited homology (∼2 % query cover) to Streptomyces phages Vanseggelen and Omar, with identities of 72.32 % and 72.66 %, respectively. A detailed comparative genome analysis, illustrated in Fig. 7C, revealed that Ph_Salpro1 and Ph_Salpro4 exhibit extensive collinearity with minimal genomic rearrangements, indicated by a large block of sequence similarity covering nearly the entire genome. Among identified prophages, Ph_Salpro3 and Ph_Alpe did not align with any known phage sequences in the database, suggesting that it represents a potentially novel prophage.

For the other prophages, BLASTn identified references with associated metadata. Ph_Ocea showed similarity to a metagenome-assembled genome (MAG) and was classified as a temperate phage within the *Bielevirus* genus, with *Aeromonas media* predicted as its host. As depicted in Fig. 7A, Ph_Ocea and Genome12_Sample2 shared multiple conserved regions with strong synteny across their genomes, further supporting this relationship. Similarly, Ph_Sapr1 was closely related to the temperate *Peduovirus* MSP0187 and was predicted to infect *Salmonella*. This comparative genomic analysis, shown in Fig. 7B, demonstrated that despite the overall conservation, Ph_Sapr1 shared homology with MSP0187 only in specific regions, suggesting divergence across other genome sections. In contrast, Ph_Sapr2 aligned with the Escherichia phage PhiR12_3, but the very low query cover (1 %) and absence of metadata for the reference sequence indicated a distant relationship; subsequent analysis confirmed its temperate lifestyle via CHERRY.

### 3.8 Predicted CAZymes, RBPs, and AMGs

Among the eight analyzed prophages, only three possess CAZyme genes, indicating that the capacity for extracellular polysaccharide degradation is sporadically distributed across the collection. Ph_Sapr1 and Ph_Sapr2 each encode a single GH24 family. Also, Ph_Salpro2 has the GH18 gene. The remaining five prophages lack detectable CAZyme annotations altogether.

Moreover, among the identified phages examined, just one yielded confidently predicted protein-coding sequences that could be assigned putative functions as RBP. Ph_Ocea offers the C-terminal half of a tail-fiber protein (gp53-like) possessing “MANVDVPSIYHTANKPSNADVGLSNLQNWSYSHSYTDATGGATKYASGKAVADAYNALNSSKLNAASYTAADVLAKLKTVGGAASGLDADLLDGQHGDYYRAWANLTGVPAAFPPSSHAHTWTSITGKPATFPPSSHSHPWSEVTGKPATYPPANHGHSWEEITGKPELFNPTGETFADGSGWTVIGKVNTPNGVKTMILQWGWARGMSGPGTTKYITFPIAFNCPVEQIHAQGTGYPWNTSGGTSSVINVAQIHSVTTVGMRVQDGDTDSTTFDISWEATGYI” amino acid sequence. No sequences were obtained for Ph_Alpe, Ph_Salpro1, Ph_Salpro2, Ph_Salpro3, Ph_Salpro4, Ph_Sapr1, or Ph_Sapr2. While some putative AMGs were identified by eggNOG-mapper and InterProScan, none of them were approved via the KEGG database with the mentioned thresholds.

### 3.9 Identified CRISPR-Cas systems

We mined 38 publicly available genomes for CRISPR-Cas signatures and obtained clear evidence for CRISPR-based immunity in 35 of them. The remaining 3 assemblies lacked either CRISPR repeats or Cas genes and were therefore considered non-CRISPR strains. Among the CRISPR loci-positive set, the number of CRISPR loci per genome ranged from one to 15, with the *S*. *proteolyticum* IBRC-M 10908 carrying the highest burden. Spacer counts paralleled locus number but were generally higher, peaking at 48 in the *O*. *halophilus* genome and exceeding 40 in several *M. persicus* strains as well as *S. proteolyticus*, *O*. *halophilus*, *N*. *halotolerans*, and *L*. *halophila* strains.

**Fig. 8:**
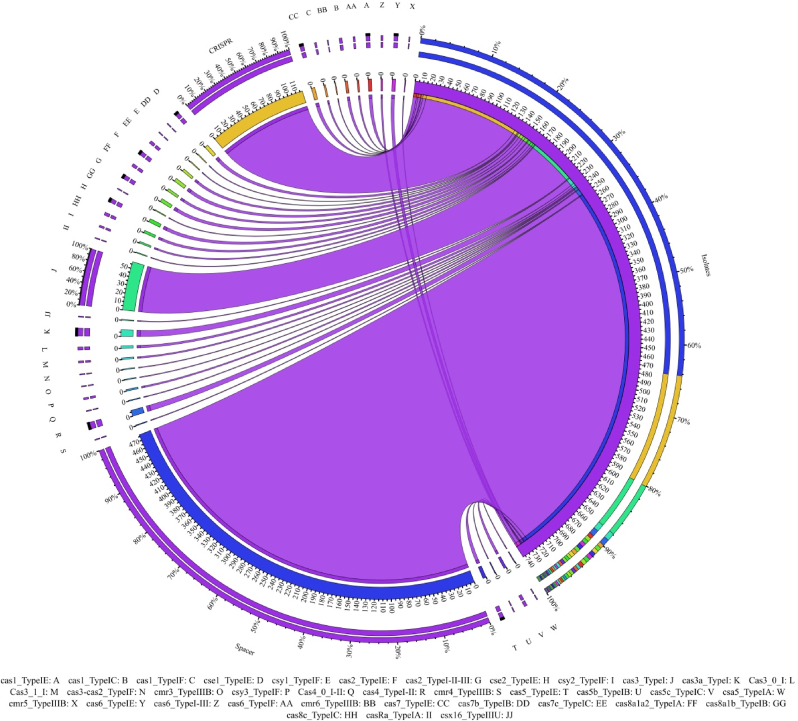
Distribution of Cas types among bacterial isolates. Cas type names are shown in English words with abbreviations indicated in the figure. This visualization uses ribbons to connect bacterial groups to their CRISPR features. The size of the inner arc shows the absolute count per group, and the outer arc shows that group's share of the total count for all groups.

Across the identified Cas genes, Cas3_TypeI emerged as the single most abundant effector, contributing 58 of the 148 Cas genes detected (39.18 %). This universal nuclease was accompanied by eight Cas3a_TypeI and eleven further Cas3 isoforms (Cas3_0_I, Cas3_1_I, and Cas3-Cas2_TypeIF), together accounting for more than 50 % of all Cas annotations. When the dataset was resolved into complete modules, the Type I-E cohort formed the largest cohort. These Cas types consist of 7 types, including Cse1, Cse2, Cas1, Cas2, Cas5, Cas6, and Cas7. Distribution of Cas types among bacterial isolates is displayed in Fig. 8.

### 3.10 Putative ACR proteins among predicted prophages

The analysis of putative ACR proteins across 8 phage genomes was conducted using AcrHub, which employs PaCRISPR and AcRanker as prediction tools. Any protein whose score exceeded the recommended threshold of 0.5 was labeled “Yes” by PaCRISPR. AcRanker's default cut-off is −5.0, so values greater than this limit were likewise classified as “Yes”.

A total of 122 protein sequences were evaluated (available in Supplementary Material 3). Overall, PaCRISPR predicted 81 proteins as potential ACRs, while AcRanker identified 59. Both tools agreed on the classification for 18 proteins, representing a substantial overlap in predictions. Ph_Alpe have the highest number of putative ACR proteins with 25 protein sequences. With 6 sequences, Ph_Ocea has the lowest ACRs among predicted prophages. These computational predictions provide a foundational set of candidate ACRs for further experimental validation.

### 3.11 Defense and anti-defense systems

Based on the comprehensive analysis of defense and anti-defense systems within the provided genomic dataset, a diverse arsenal of bacterial immune mechanisms was identified. A total of 323 defense systems were found in bacterial strains. Among bacterial genomes, *S. proteolyticus* strain TGB10 and *M. azerbaijanicus* have the highest defense system (20 and 19 defense systems respectively). Restriction-modification systems (RM), particularly Type I was among the most commonly identified (15.17 % prevalence). Other frequently detected systems include Ceres (5.57 % prevalence), AbiD, RM_Type_IIG and RM_Type_IV (3.71 % prevalence).

Concurrently, anti-defense systems, which serve to counteract bacterial immunity, were also identified in a subset of the genomes. In total, 5 anti-defense systems were identified in bacterial genomes. The most common anti-defense system detected was ardc (40 % prevalence), found in *Alteribacillus* species. Other anti-defense systems like apyc1, mom, and Adnd_p0020_p0021 were present in fewer instances (20 % prevalence each).

The analysis was extended to predicted prophages within the genomes, uncovering that these viral elements also carry genes encoding for both defense and anti-defense systems. Notably, some prophages were found to possess RM type II and IV systems (4 and 1 prophages), and Butters_gp30_gp31 (2 prophages). Also, dGTPase, PARIS_II_merge, and Sirona systems were identified just once. Moreover, the anti-restriction system ardc was identified in Ph_Haka2 and Ph_Haka3. The complete, detailed listings of all defense and anti-defense systems for each bacterial strain and prophage are available in Supplementary Material 4.

### 3.12 Genomic architecture of integrated metabolic-defensive islands

A comprehensive analysis of genomic co-localization was performed across twelve bacterial strains, revealing 23 distinct genomic islands in total. These islands were categorized based on their primary associated features: 17 defense-metabolite islands, 4 CRISPR-metabolite islands, 1 Cas-metabolite island, 1 combined CRISPR-Cas-defense-metabolite island, and 1 prophage-metabolite island. The number and complexity of islands varied considerably between strains, with *L. halophila* and *O. limi* representing the most architecturally complex cases, each harboring four distinct islands. The detailed information of each island is provided in Supplementary Material 5.

### 3.13 Defense-metabolite islands

Defense-metabolite islands constituted the majority of identified regions, demonstrating a prevalent genomic architecture where BGCs are physically linked to diverse bacterial defense systems. These islands displayed a range of integrations, from simple adjacency to complex interweaving of metabolic and defensive genetic modules. For example, in *H. lysinitropha*, two hybrid islands were identified, one contained a Type I RM system alongside an azole-containing RiPP and a betalactone BGC, and another one contained a type III RM and a sactipeptide module. *M. persicus* 23561platanus featured an island co-localizing an RM Type I system, an AbiD abortive infection module, and an ectoine BGC, suggesting a multifunctional genomic region. *M. azerbaijanicus* also contained a defense-metabolite island pairing an NI-siderophore BGC with an RM Type II system. The strains *S. proteolyticus* DSM19052 and DSM8285 each possessed an island pairing a betalactone BGC with a Belisama defense system. *Oceanimonas* sp. GK1 also exhibited this architecture, with an NI-siderophore BGC (746,012–777,519) adjacent to a Belisama defense system (804,690–805,508). The consistent finding across multiple strains indicates a common genomic strategy for coupling secondary metabolism with innate immunity.

### 3.14 CRISPR and cas-metabolite islands

Islands containing CRISPR arrays or Cas gene clusters in proximity to BGCs were also identified. *A. persicus* M10081 and *A. bidgolensis* DSM 22560 each possessed CRISPR-metabolite islands, where a CRISPR array was located near a lanthipeptide or betalactone BGC, respectively. In *O. limi*, a more complex arrangement was observed: A Cas gene cluster was found adjacent to a T3PKS BGC, forming a Cas-metabolite island, while a separate CRISPR array was located near a lanthipeptide-class II BGC. *S. halophila* also exhibited a CRISPR-metabolite island featuring a CRISPR array next to an NI-siderophore cluster. These associations suggest potential functional or regulatory interplay between adaptive antiviral defense mechanisms and metabolic pathways.

### 3.15 Prophage-metabolite island

A single prophage-metabolite island was identified in *S. proteolyticus* IBRC-M 10908K. In this architecture, a lanthipeptide-class-III BGC (3,415,756–3,438,299) is located in close proximity to the prophage Ph_Salpro4 (3,477,714–3,537,879).

### 3.16 A multi-layered adaptive immunity and metabolic island in *S. proteolyticus* TGB10

A particularly complex and noteworthy genomic architecture was identified in *S. proteolyticus* TGB10, creating a combined CRISPR-Cas-defense-metabolite island. This single, extensive region assembled multiple adaptive and innate immune components with a specialized metabolic cluster. The island is anchored by a CRISPR array (657,024–657,967) immediately adjacent to a full Cas gene cluster (658,046–666,649). Downstream, two linked Hachiman defense system components (HamB and HamA_2) were identified (676,953–680,635). Furthermore, the island encodes a hydrogen-cyanide BGC (705,616–718,582). This island represents a highly integrated configuration, directly linking the genetic machinery for adaptive immunity (CRISPR-Cas), innate defense (Hachiman), and hydrogen cyanide production within one genomic segment.

### 3.17 Notable cases of fully integrated architectures: *L. halophila* and *O. limi*

**Fig. 9:**
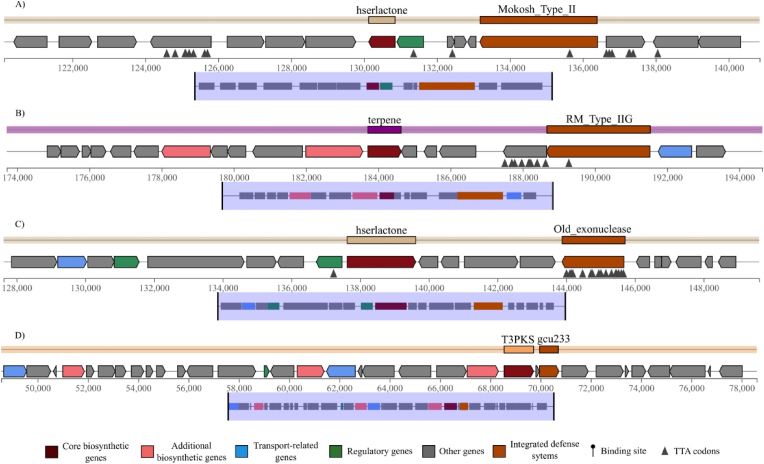
Representative examples of fully integrated defense-metabolite genomic islands in *L. halophila* and *O. limi*. (A) In *L. halophila*, a hserlactone BGC is precisely integrated with the Mokosh_TypeII defense system. (B) A separate terpene BGC in *L. halophila* is fully embedded within an RM_Type_IIG defense system, sharing the identical genomic locus. (C) A second hserlactone island in *L. halophila* shows fusion with an Old_exonuclease defense system. (D) In *O. limi*, a T3PKS BGC completely embeds the defense system gcu233.

*L. halophila* and *O. limi* presented the most intricate and fully integrated genomic organizations, meriting specific attention (see Fig. 9). In *L. halophila*, four defense-metabolite islands were characterized by deep functional overlap. For instance, a hserlactone BGC was precisely integrated with the Mokosh_TypeII defense system, where the genomic segment ctg3_124 was concurrently identified as part of both the biosynthetic and defensive operons. A second hserlactone island was fused with an Old_exonuclease system. Furthermore, a terpene BGC was fully embedded with an RM_Type_IIG system, sharing the identical genomic locus (ctg4_181). An additional island contained a terpene BGC alongside a PD-Lambda-5 system.

Similarly, *O. limi* displayed four highly composite islands. One defense-metabolite island contained a lanthipeptide-class-III BGC that is close to three distinct defense systems: Ceres, RM_Type_IIG, and RloC. Another island featured a T3PKS BGC within which the defense system gcu233 was completely embedded, with the segment ctg31_73 representing the exact overlap between the metabolic and defense annotations. This strain also contained the aforementioned Cas-metabolite island and a CRISPR-metabolite island.

## 4 Discussion

Our genomic survey of native Iranian aquatic bacteria reveals that these isolates are equipped with a sophisticated genetic arsenal, comprising a rich suite of secondary metabolites and bacteriocins, a collection of host-specific prophages, and robust bacterial defense systems. This genetic combination underscores a multifaceted evolutionary strategy for thriving in extreme environments.

In total we identified dozens of BGCs spanning diverse chemistries. Most striking was the ubiquity of ectoine biosynthesis. An ectoine operon appeared 41 times across the genomes, underscoring its critical role for survival in high-salinity environments. Other common BGCs included terpenes and Type III polyketide synthases (17 of each), as well as betalactones, siderophores, pigments (melanin, resorcinol), and various small-peptide pathways. For example, *L*. *halophila* alone encoded nine distinct BGCs. Intriguingly, we also found numerous hybrid clusters (e.g. contiguous arylpolyene–resorcinol modules in *M*. *persicus*, and NRPS–metallophore hybrids in *M*. *iranensis*), suggesting these bacteria can synthesize novel “mosaic” metabolites. Such breadth of secondary metabolism is consistent with observations from other extreme habitats. For instance, metagenomic surveys of Atacama Desert soils also uncovered hundreds of novel BGCs (dominated by NRPS, RiPP and terpene types) in uncultured bacteria [53].

These secondary metabolites likely have **important ecological roles**. For example, ectoine is a compatible solute synthesized by bacteria in response to high osmolarity. Microorganisms can also take up ectoine from the environment for osmostress protection. In *Virgibacillus pantothenticus*, ectoine serves as a key protectant against high salinity and its production is also induced by cold stress, providing protection against low temperatures [54]. Pigmented terpenes or polyketides may protect cells from UV or oxidative damage [55], and siderophores would help scavenge scarce iron [55]. The presence of multiple BGCs per genome suggests these bacteria can tailor their chemistry to microhabitat conditions. This is consistent with the view that extremophiles “produce different repertoires of bioactive metabolites” than mesophiles [56]. Indeed, recent work emphasizes that organisms from unusual niches often carry cryptic or novel BGCs that mediate niche-specific adaptations [53]. From a biotechnology perspective, these findings are equally exciting. Many of the detected pathways encode compounds of applied value. For example, ectoine itself is a high-value osmolyte used in cosmetics and biotech as a stress-protectant [57].

We also uncovered an extensive diversity of bacteriocin gene clusters. BAGEL4 predicted dozens of ribosomally-synthesized peptide antibiotics in these genomes. Many RiPP classes were represented (e.g. sactipeptide clusters, lasso peptides, lantibiotic-like RiPPs, etc.), and in several cases the core peptide gene was identified. Notably, we found clusters for known bacteriocins including Linocin_M18, Michiganin-A, Zoocin_A, and single instances of Colicin and Closticin_574. This spectrum from Gram-negative colicin to streptococcal Zoocin and clostridial Closticin illustrates that these isolates carry a broad arsenal of antimicrobials. In microbial communities, such bacteriocins serve as potent competition weapons as natural antibiotics, which allow producers to inhibit or kill closely related strains and secure resources [58]. Indeed, the importance of these peptides in salt-loving microbes is documented (e.g. the halophile *Staphylococcus simulans* produces the lantibiotic nukacin that inhibits *Bacillus subtilis* [59], and haloarchaea secrete “halocins” active against other archaea [60]). By analogy, our isolates’ bacteriocins likely shape community composition in brine pools and sediments by excluding rivals or modulating microbial diversity. From an applied perspective, such extremophile-derived peptides might have novel targets or stability profiles, and could be developed as new narrow-spectrum antibiotics or food biopreservatives.

Beyond their native defenses and chemical weapons, these bacteria serve as reservoirs for a diverse and host-specific community of prophages. Our analysis identified eight high-quality prophages belonging to several taxonomic lineages, all of which were unique to their host species. These prophages are not mere genetic hitchhikers; they encode auxiliary genes that may directly influence host ecology. For instance, we identified CAZymes such as glycoside hydrolases (GH24) and even a chitinase (GH18), as well as RBP and candidate ACR factors. Phage-borne CAZymes such as GH24 [61] often function as endolysins or polysaccharide-degrading enzymes. The identification of CAZymes suggests a potential role in degrading the host's cell wall or environmental polysaccharides. This could aid in infection initiation by exposing bacterial receptors or provide a metabolic advantage to the lysogenized host in carbon-rich environments like wetlands and hypersaline lakes.

The in silico prediction of a GH18 gene within the Ph_Salpro2 prophage in our study is particularly intriguing. This finding can be insightfully discussed in the context of the recent study by Middelboe et al. [62]. They described a system where the bacterial host encoded a GH18 chitinase, while its prophage provided a complementary GH19 chitinase. They demonstrated experimentally that this prophage-encoded enzyme was functional, significantly enhancing the host's chitin degradation capacity and growth, and its expression was strategically regulated by substrate availability. Our discovery suggests a different, yet equally compelling, evolutionary strategy. The phage may be augmenting the host's existing enzymatic capability by providing an additional copy of a similar enzyme (GH18) rather than a novel one (GH19). The experimental framework established by Middelboe et al. [62] is a direct pathway for future validation of our predictions. Functionally characterizing these prophage-encoded GH18 genes will be essential to confirm if they operate as collaborative AMGs that enhance host fitness, as selfish genes that aid in phage infection, or as a combination of both, thereby critically shaping the ecology of these unique Iranian bacterial isolates.

Furthermore, several small prophage proteins were predicted to be *anti*-CRISPR candidates. This fits into the known arms race that phages regularly evolve ACR proteins to inhibit host CRISPR-Cas systems [63], enabling infection despite bacterial immunity. Moreover, these prophages carry putative auxiliary and counter-defense genes, suggesting a potential, though limited, functional impact. The host genomes themselves encode abundant anti-phage systems, notably CRISPR-Cas. We observed CRISPR loci in 34 of the 38 strains, with Type I systems (particularly I-E subtype) and associated Cas3 helicases especially common. This is consistent with the fact that Class 1 CRISPR (multi-subunit Type I) comprises ∼90 % of CRISPR loci and that Type I is the most widespread subtype in bacteria [64]. The prevalence of Type I-E CRISPR-Cas systems, underscored by a notable abundance of the Cas3 helicase-nuclease, is a key indicator of robust adaptive immunity in these isolates. This strong genomic defense likely drives selection for phage countermeasures; the presence of ACR candidates in prophages matches prior observations that phages evolve *anti*-CRISPR proteins to overcome such immunity [63]. In addition to CRISPR, the bacterial genomes also likely harbor other defense islands (restriction-modification, abortive infection, toxin–antitoxin, etc.) in line with general anti-phage strategies [63], and conversely, some prophages carried predicted anti-defense genes. Together, this co-occurrence of host defense systems and prophage counter-defenses illustrates the dynamic co-evolution between phages and bacteria in these strains.

The discovery of these prophages and genes has significant ecological implications for the extreme Iranian environments from which the hosts derive. Prophage-carried CAZymes could enhance microbial fitness under harsh conditions. For example, phage-encoded chitinase (observed in one prophage) has been shown in other studies to boost host carbon acquisition and growth on chitin, aiding adaptation in nutrient-limited environments [62]. More broadly, phages in physicochemically stressed habitats often carry unique metabolic genes that modulate host nutrient use and stress tolerance, thus influencing biogeochemical cycling [65]. In the hypersaline contexts of Iran, such prophage genes might help bacteria tolerate osmotic stress or exploit unusual substrates. The widespread CRISPR-Cas immunity likewise shapes community composition; phage predation can limit host populations (the “viral shunt”), while ACR-bearing phages may allow lysogens to persist. These interactions likely influence ecosystem processes, as temperate phages in similar studies are known to contribute to microbial genetic diversity and resilience under stress [62,65].

The most significant theoretical contribution of this study is the characterization of the Multi-Functional Genomic Island, for which we propose the “Fortress Hypothesis” to explain its underlying architecture. This hypothesis arises from the observation that the production of secondary metabolites is metabolically expensive for a bacterium [66]. This substantial investment renders the producing cell a high-value target for both “cheater” strains, which utilize the metabolite without producing it [67], and for phages that exploit metabolically active hosts [68]. Consequently, a powerful selective advantage exists in physically coupling the BGCs responsible for production with the genetic systems responsible for protection. This linkage is a defining feature of these genomic islands.

Protection manifests in several integrated ways. Against phage lysis, the placement of a defense system adjacent to a biosynthetic cluster ensures that any phage attempting to infect the cell will trigger a defensive response. In cases involving abortive infection systems, this may result in the sacrificial death of the infected cell [69], thereby halting viral replication to protect the clonal population and preserve the valuable biosynthetic capacity within surviving kin. Furthermore, these architectures contribute to the stabilization of the mobile genetic elements on which many biosynthetic and defense genes are located. The presence of systems like CRISPR-Cas or Hachiman on such an island can act as a “selfish” addiction module, preventing its excision or loss and thereby incidentally benefiting the host.

Based on the genomic architectures observed in this study, particularly the discovery of anti-defense phage genes embedded within metabolite clusters and the notable abundance of TTA codons within adjacent defense system genes (as exemplified in the *L. halophila* hserlactone/Old_exonuclease island in Fig. 9C), we propose an extended hypothesis for regulatory integration. We hypothesize that beyond simple physical linkage, these multi-functional islands employ a shared, resource-dependent regulatory checkpoint. The high frequency of the rare TTA codon (decoded by the scarce Leu-tRNA^*TTA*^) within key defense genes suggests that the expression of the protective apparatus is directly coupled to the translational capacity of the cell. Concurrently, the expression of many BGCs is also known to be sensitive to the availability of this same tRNA [70]. Therefore, the activation of both secondary metabolite production and antiviral defenses may be gated by a common cellular signal: the abundance of the Leu-tRNA^*TTA*^, which itself is tied to the metabolic and physiological state of the cell. This creates a fail-safe mechanism where the costly commitment to chemical production and the induction of defensive systems are co-regulated, ensuring that defense mobilization occurs only when the translational machinery is fully competent and the cell is primed for metabolically expensive processes. Finally, observed gene fusions suggest a potential for regulatory coupling. It is plausible that the environmental signals triggering secondary metabolism, such as quorum sensing at high population density, simultaneously upregulate adjacent antiviral defenses. This “panic button” strategy would ensure a cell is most heavily defended precisely when it becomes most visible to predators and is at its peak metabolic activity.

Beyond ecology, our findings point to biotechnological potential. Phage-derived CAZymes and lytic enzymes (e.g., GH23/24 lysozymes or other enzymes displayed in Supplementary Material 2) are promising antimicrobials (enzybiotics) against pathogens [71,72]. Furthermore, novel ACR proteins may inform the development of programmable CRISPR inhibitors or expanded genome-engineering tools. Thus, the unique prophage genes uncovered here merit future exploration for industrial and medical applications.

In summary, the newly identified prophages, secondary metabolites, and bacteriocins collectively paint a picture of highly adapted microbial residents. Rather than being passive, these microbes maintain a dynamic genetic equilibrium; They produce osmolytes and antimicrobials to manage abiotic and biotic stress, while simultaneously engaging in an evolutionary tug-of-war with their resident prophages. This interplay likely fine-tunes their adaptation to the specific challenges of Iranian hypersaline lakes and wetlands. From a biotechnology standpoint, this microbial collection represents an untapped trove of potential, offering novel enzyme candidates (CAZymes), CRISPR inhibitors (ACRs), bioactive peptides (bacteriocins), and stress-protectant pathways (ectoine). Future work focusing on the experimental validation and heterologous expression of these predicted genes and clusters will be essential to unlock their full applied value.

This study has certain limitations. The prophages and their putative gene functions were identified solely through in silico bioinformatic tools, and the secondary metabolite clusters remain predictions. Experimental validation is therefore essential to confirm activity. Nonetheless, by integrating prophage analyses with genome mining for biosynthetic clusters, this work provides a foundational genomic blueprint. Its true value will be realized through functional validation of the identified ‘hot spots' to unlock their biotechnological potential. To capitalize on this potential, critical next steps should focus on validating ectoine biosynthesis and the antimicrobial activity of RiPPs, confirming the function of the prophage-encoded CAZyme GH18 to understand its ecological role, and characterizing the predicted *anti*-CRISPR proteins to advance CRISPR-Cas tool engineering. Such wet-lab studies will be crucial to move beyond genomic prediction and harness the unique adaptations encoded within these Iranian extremophile strains.

## 5 Conclusion

This first genomic survey of native Iranian aquatic bacteria reveals a rich genetic arsenal strategically organized into multi-functional genomic islands that co-localize BGCs with defense and anti-defense systems. We propose the “Fortress Hypothesis” to explain this architecture: the physical coupling of metabolically expensive secondary metabolite production with protective systems (e.g., CRISPR-Cas, restriction-modification) safeguards cellular investment against phage predation and genetic loss.

These findings underscore a sophisticated co-evolutionary landscape where chemical innovation and antiviral immunity are genomically and functionally intertwined, fine-tuning adaptation to extreme Iranian habitats. Beyond ecology, this resource offers significant biotechnological potential, including novel enzymes (e.g., prophage CAZymes), CRISPR inhibitors (ACRs), antimicrobial peptides (bacteriocins), and stress-protectant pathways (e.g., ectoine). The identified genomic islands provide prioritized targets for future experimental validation and applied development.

## Declaration of competing interest

The authors declare that there is not any conflict of interest.

## Data Availability

Data will be made available on request.
