# Supplementary material for: In silico exploration of the genomic repertoire of Iranian aquatic bacteria: Prophage carriage, bioactive compound potential, CRISPR-Cas immunity, and integrated defensive-metabolic islands
Source: Biochem Biophys Rep. 2026 Jan 28;45:102452. doi: 10.1016/j.bbrep.2026.102452 (PMC12874462; doi:10.1016/j.bbrep.2026.102452)
Supplement: Multimedia component 2 [file mmc2.docx]

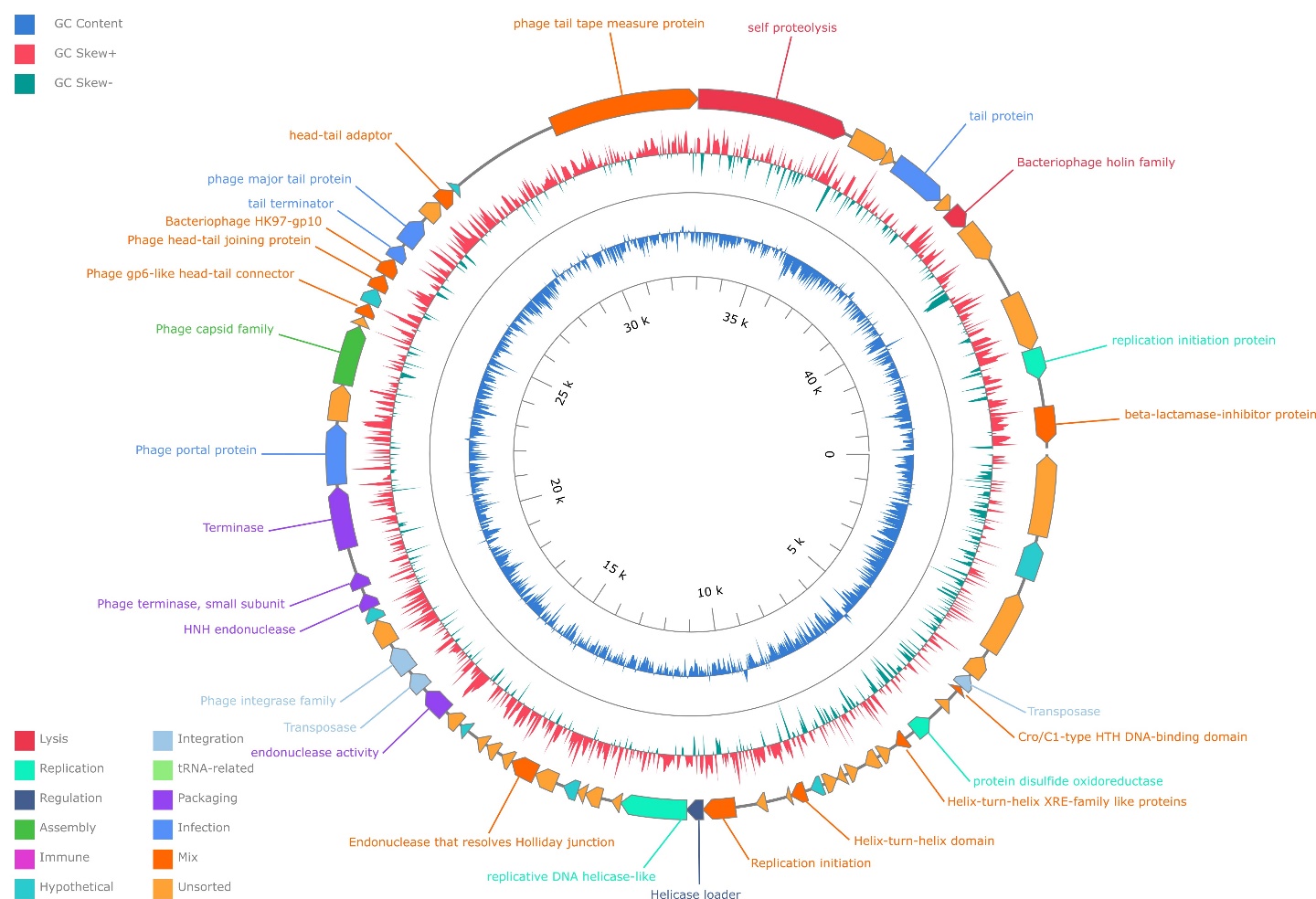


**Fig. S1.** Circular genome map of the prophage Ph-Alpe.


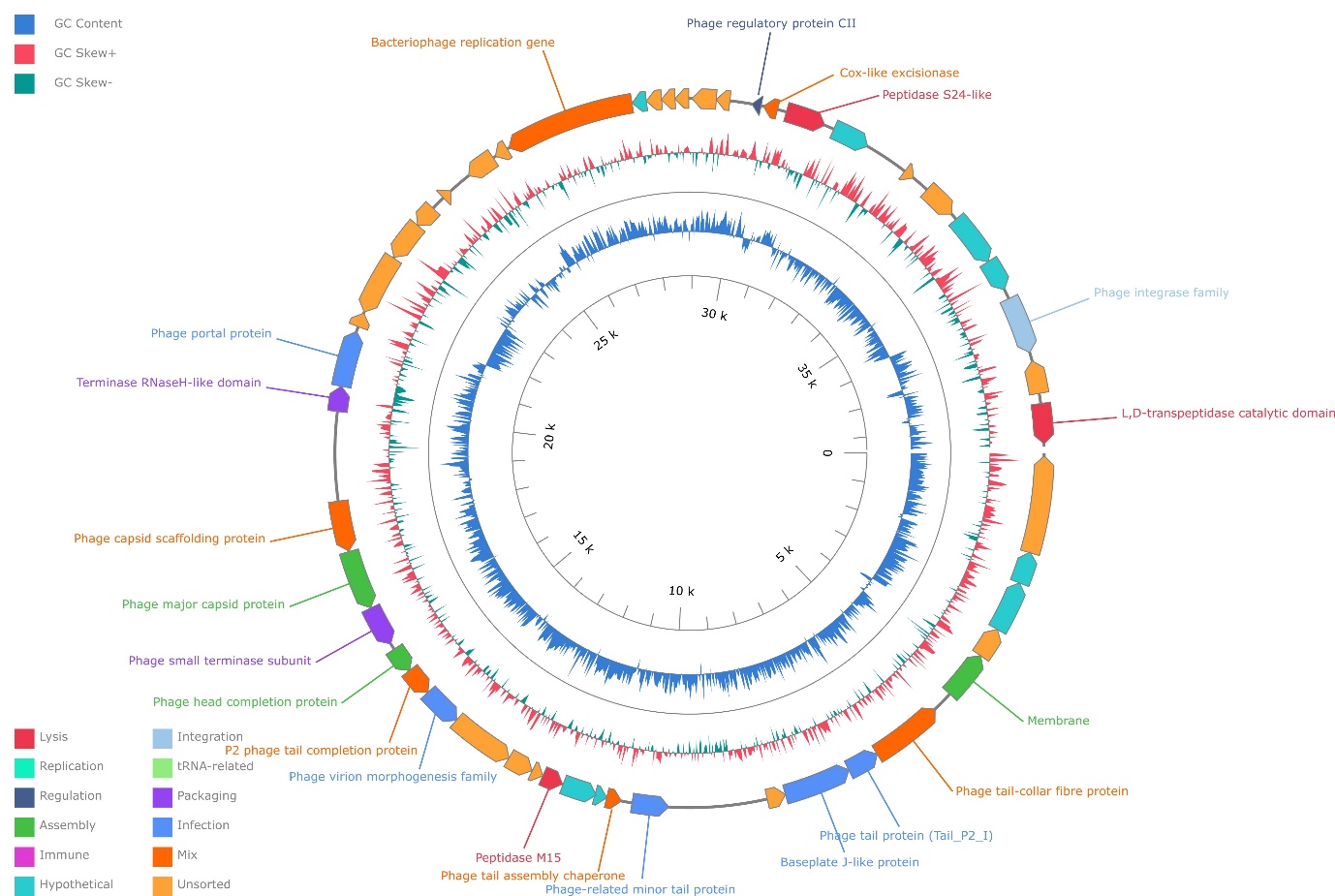


**Fig. S2.** Linear genome maps of the prophage Ph-Ocea.


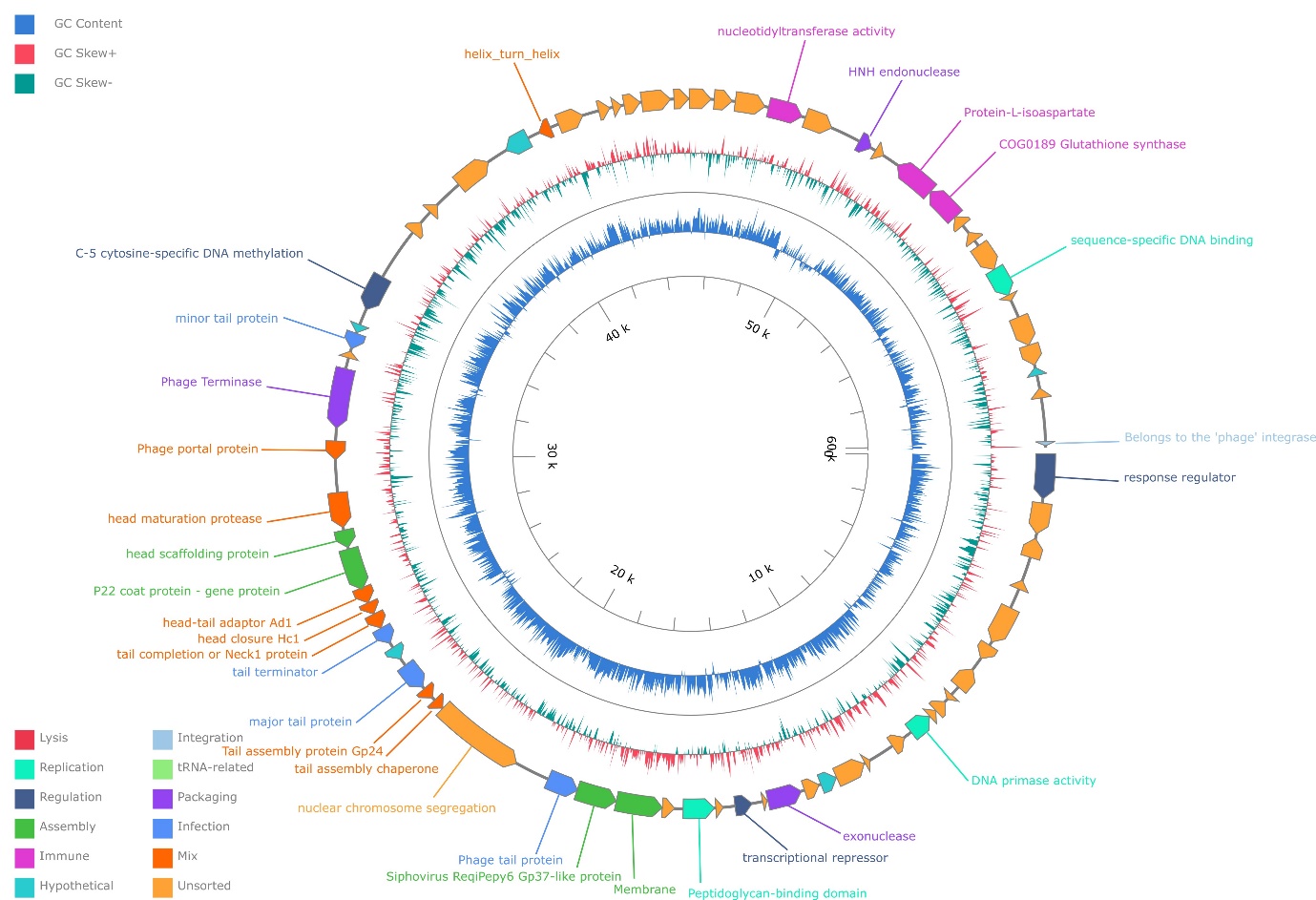


**Fig. S3.** Linear genome maps of the prophage Ph-Salpro1.


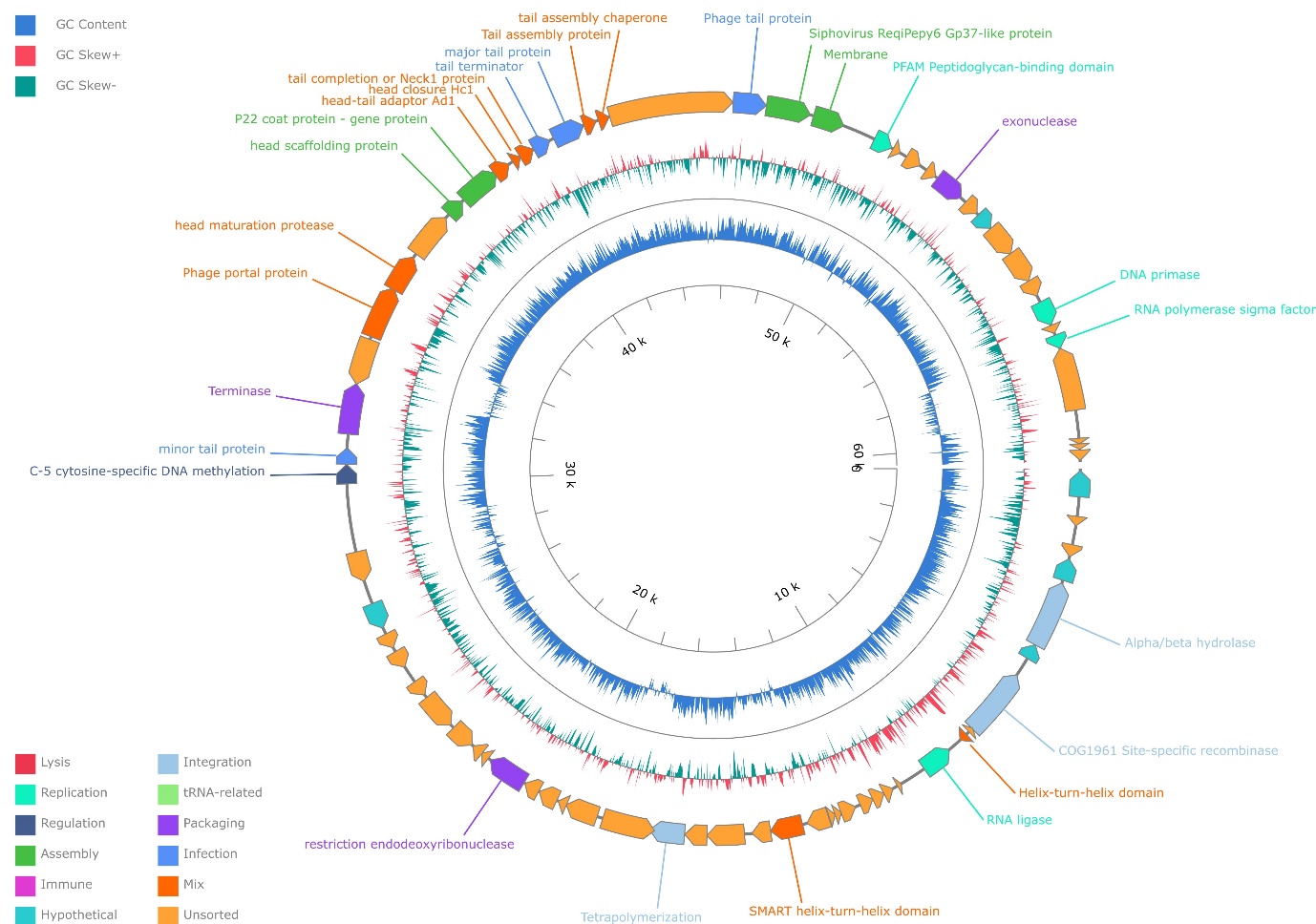


**Fig. S4.** Linear genome maps of the prophage Ph-Salpro2.


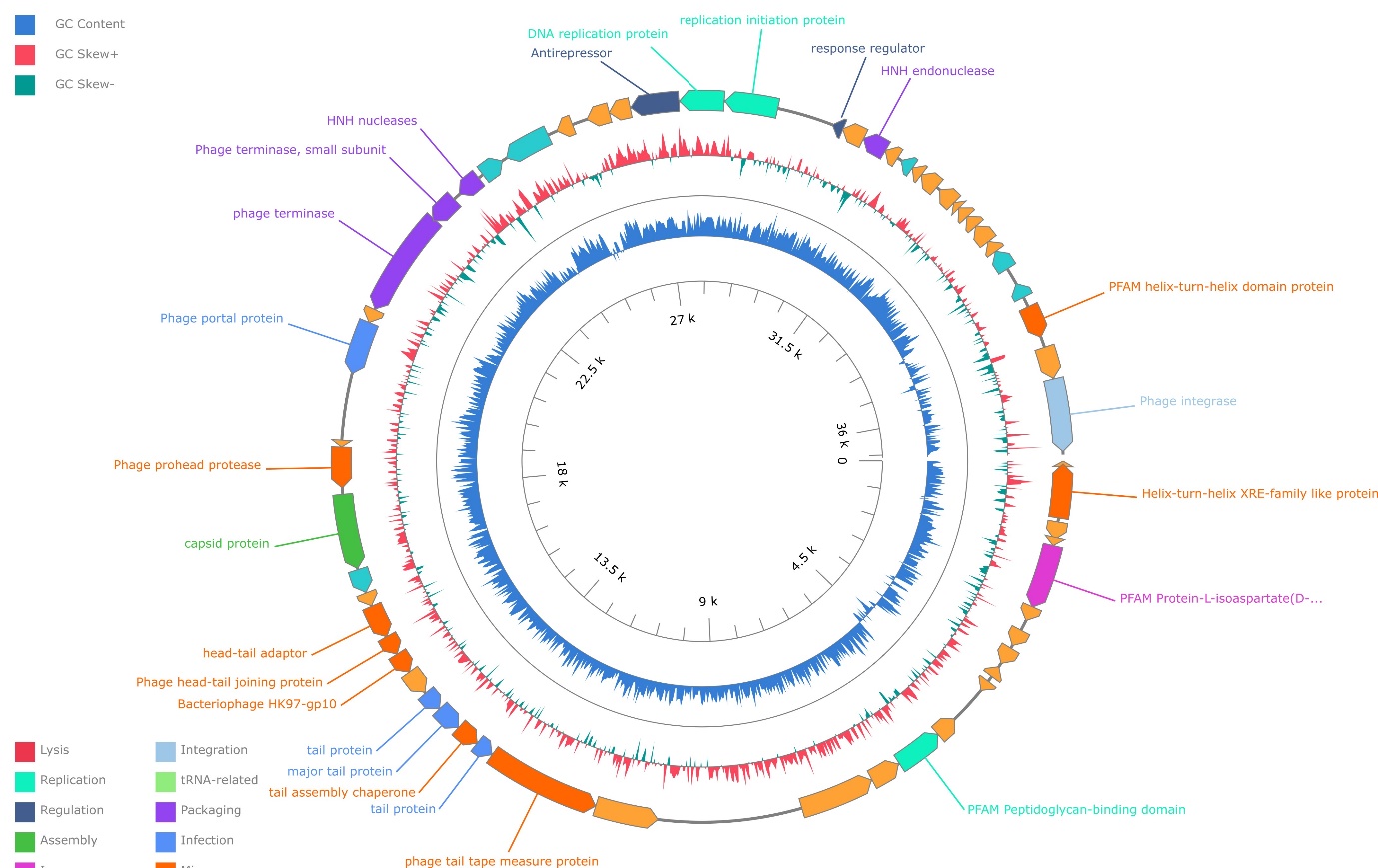


**Fig. S5.** Linear genome maps of the prophage Ph-Salpro3.


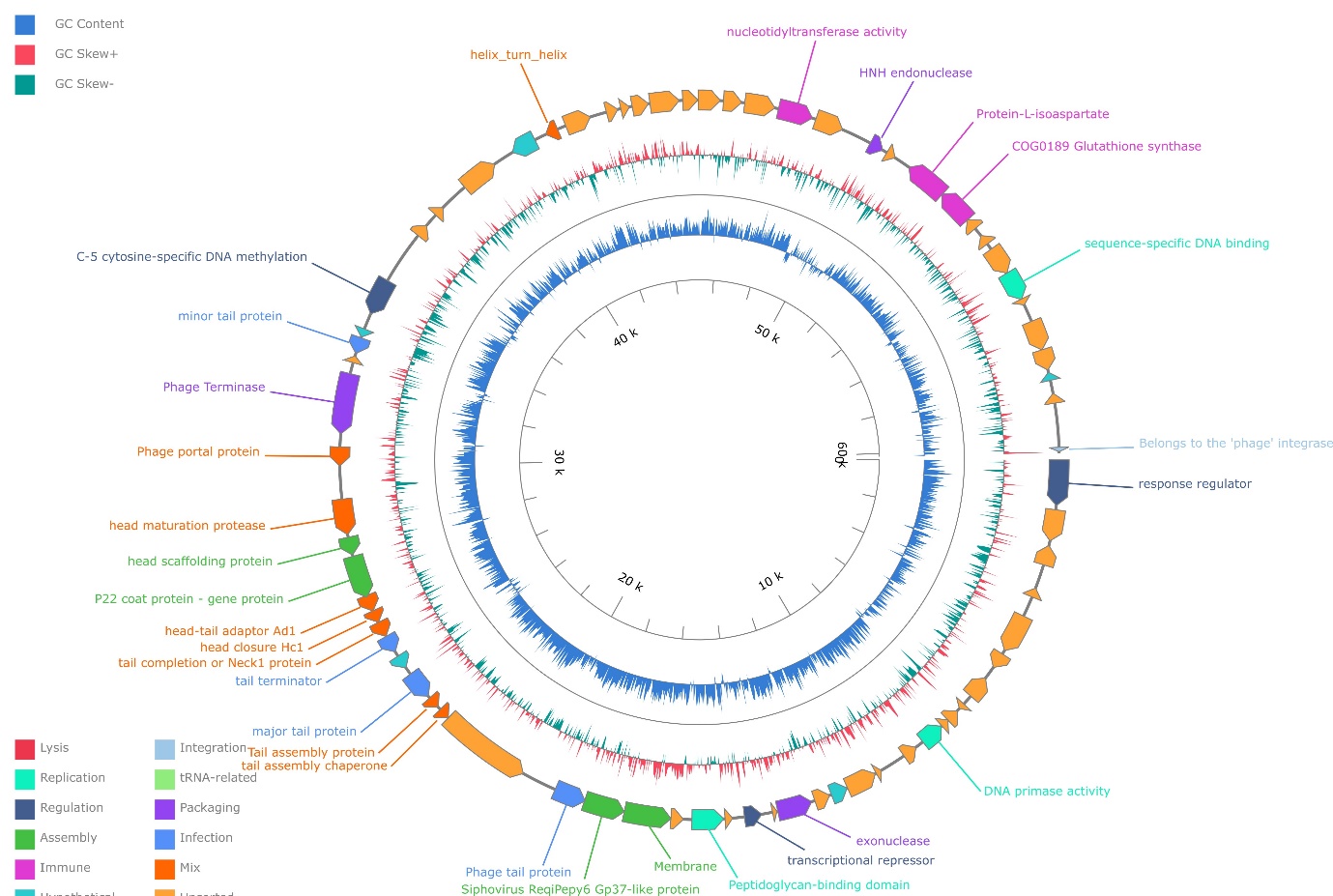


**Fig. S6.** Linear genome maps of the prophage Ph-Salpro4.


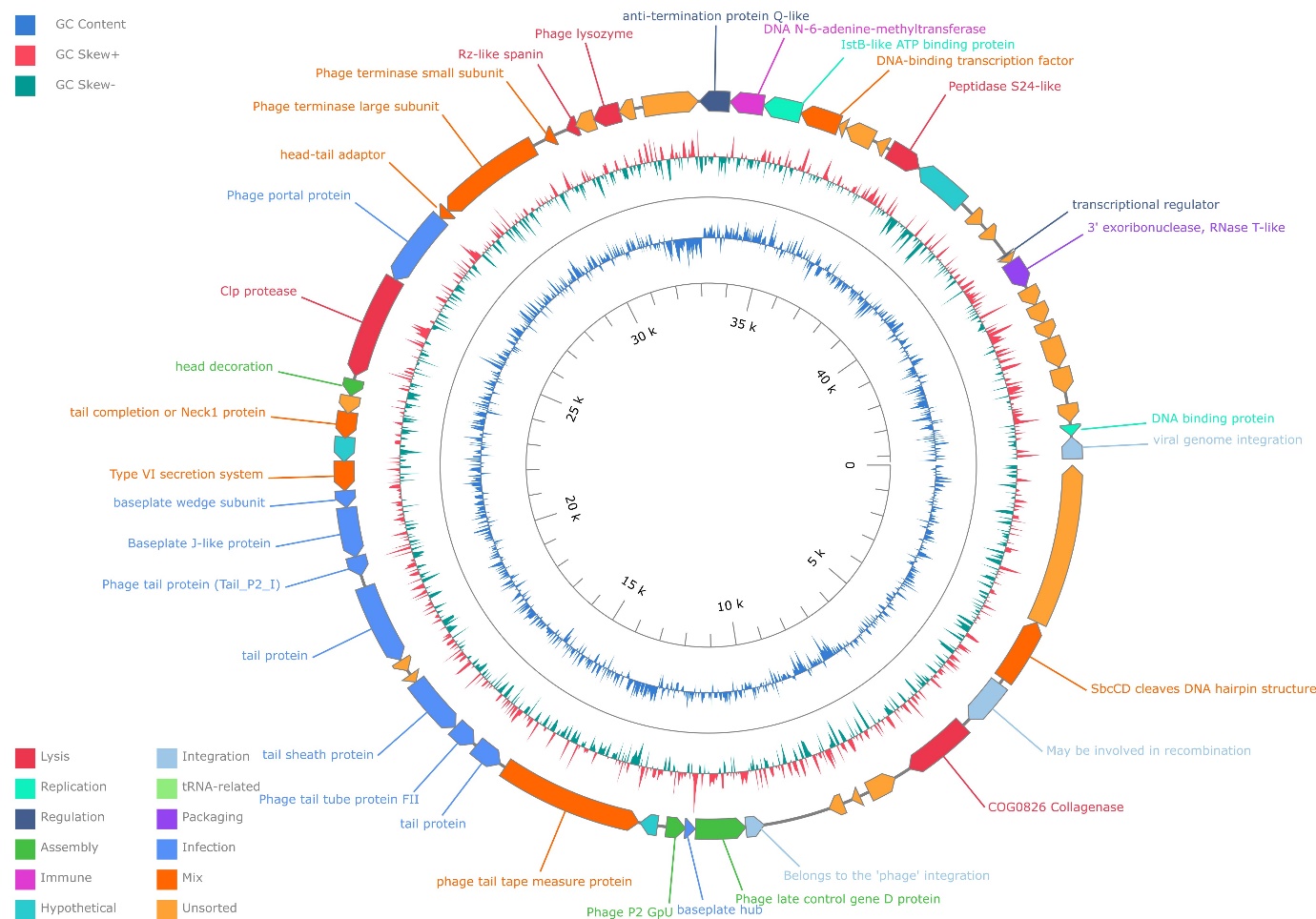


**Fig. S7.** Linear genome maps of the prophage Ph-Sapr1.


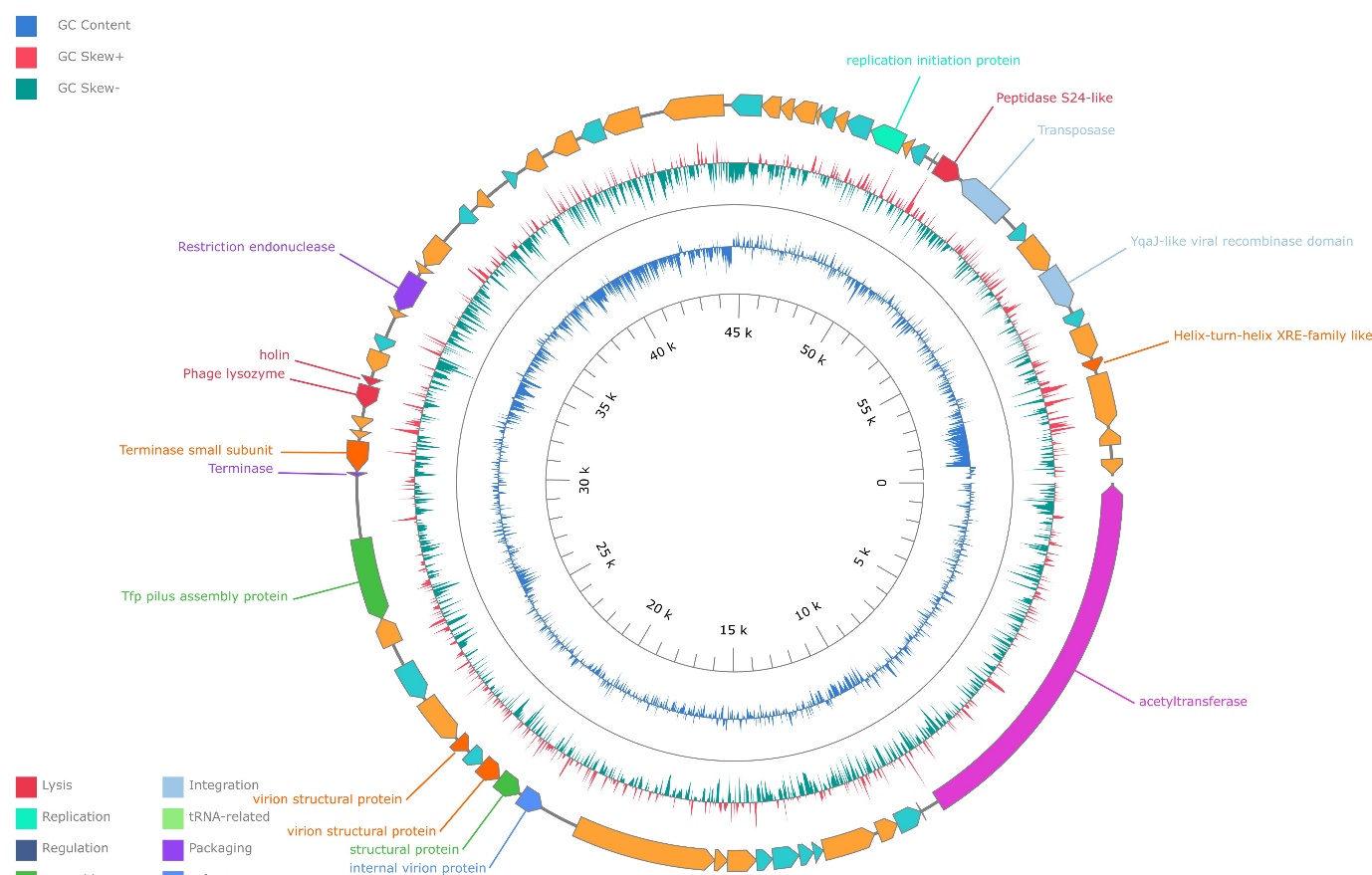


**Fig. S8.** Linear genome maps of the prophage Ph-Sapr2.
